# The effect of hand hygiene promotion programs during epidemics and pandemics of respiratory droplet-transmissible infections on health outcomes: a rapid systematic review

**DOI:** 10.1186/s12889-021-11815-4

**Published:** 2021-09-25

**Authors:** Koen Veys, Kim Dockx, Hans Van Remoortel, Philippe Vandekerckhove, Emmy De Buck

**Affiliations:** 1 Centre for Evidence-Based Practice (CEBaP), Belgian Red Cross, Motstraat 42, Mechelen, Belgium; 2 Belgian Red Cross, Mechelen, Belgium; 3 Department of Public Health and Primary Care, KU Leuven, Leuven, Belgium

**Keywords:** Hand hygiene, Hand washing, Respiratory infections, Influenza, COVID-19, Acute respiratory syndrome

## Abstract

**Background:**

Public health strategies in the context of respiratory droplet-transmissible diseases (such as influenza or COVID-19) include intensified hand hygiene promotion, but a review on the effectiveness of different ways of promoting hand hygiene in the community, specifically for this type of infections, has not been performed. This rapid systematic review aims to summarize the effectiveness of community-based hand hygiene promotion programs on infection transmission, health outcomes and behavioral outcomes during epidemic periods in the context of respiratory droplet-transmissible diseases. We also included laboratory-confirmed health outcomes for epidemic-prone disease during interepidemic periods.

**Methods:**

We searched for controlled experimental studies. A rapid systematic review was performed in three databases and a COVID-19 resource. Following study selection (in which studies performed in the (pre-)hospital/health care setting were excluded), study characteristics and effect measures were synthesized, using meta-analyses of cluster-RCTs where possible. Risk of bias of each study was assessed and the certainty of evidence was appraised according to the GRADE methodology.

**Results:**

Out of 2050 unique references, 12 cluster-RCTs, all in the context of influenza, were selected. There were no controlled experimental studies evaluating the effectiveness of hand hygiene promotion programs in the context of COVID-19 that met the in−/exclusion criteria. There was evidence that preventive hand hygiene promotion interventions in interepidemic periods significantly decreased influenza positive cases in the school setting. However, no improvement could be demonstrated for programs implemented in households to prevent secondary influenza transmission from previously identified cases (epidemic and interepidemic periods).

**Conclusions:**

The data suggest that proactive hand hygiene promotion interventions, i.e. regardless of the identification of infected cases, can improve health outcomes upon implementation of such a program, in contrast to reactive interventions in which the program is implemented after (household) index cases are identified.

**Supplementary Information:**

The online version contains supplementary material available at 10.1186/s12889-021-11815-4.

## Background

The rapid, widespread escalation of Coronavirus Disease 2019 (COVID-19) has led the WHO to declare this disease a global pandemic [1]. Previously, other respiratory infectious diseases, such as the Severe Acute Respiratory Syndrome (SARS) and Middle East Respiratory Syndrome (MERS), have caused outbreaks of epidemic proportions [2, 3]. It is clear that such epidemics have the potential to trigger a collapse of health care systems, when the limits of health care capacity are exceeded [4, 5]. Effective public health strategies, leveling off hospitalization peaks, are crucial to avert such a scenario. Intensified hand hygiene is regarded as one of the most important mitigation techniques in infection control and is still being recommended (August 2021; 1 year and a half into the pandemic) by the WHO in the context of COVID-19 [6]. Indeed, hand washing with soap and water, or hand disinfection with alcohol-based sanitizers, should – in theory – eliminate the virus from people’s hands, thereby preventing transmission when touching their eyes, nose or mouth after having touched a surface contaminated by infectious droplets [7]. This review does not aim to evaluate the effect of hand hygiene as such, but was initiated in response to the emerging COVID-19 pandemic to examine the effectiveness of community-based promotion programs for hand hygiene. We were not only interested in studies evaluating the effectiveness of such promotion programs in the context of COVID-19, but also in the context of other infectious diseases with similar transmission modes, as we anticipated a scarcity in COVID-19 controlled experimental studies at the time of our search. To our knowledge, a review of the body of evidence for the effectiveness of hand hygiene promotion programs on health outcomes, during epidemic periods of COVID-19 or other respiratory droplet-transmissible diseases, is non-existent. Therefore, we asked the following research question: during epidemics/pandemics of respiratory infections, are hand hygiene promotion programs, organized at the community level, effective compared to no such programs or other interventions to improve health outcomes? Besides controlled experimental studies performed during epidemic periods, we also aimed to include studies from interepidemic periods, for epidemic-prone diseases such as influenza, that reported laboratory-confirmed health outcomes. We hypothesized that, regardless of the period, hand hygiene promotion programs would have a beneficial effect on health outcomes, as evidenced by fewer infected people compared to the control group.

## Methods

This systematic literature review was conducted according to the PRISMA statements (see Additional file 1) [8]. No protocol for the systematic literature searches was submitted beforehand.

### Data sources and searches

We conducted a systematic literature search in three databases (The Cochrane Library, MEDLINE (PubMed interface) and Embase (Embase.com interface); search date: May 27, 2020 with an update on July 23, 2021) and the NIPH systematic and living map on COVID-19 evidence (search date: July 1, 2020 with an update on August 6, 2021) [9] to answer the following research question: during epidemics/pandemics of respiratory infections, are hand hygiene promotion programs effective compared to no such programs or other interventions to improve health outcomes?

One reviewer developed a search strategy based on search terms describing the respiratory droplet-transmissible diseases, the epidemic (or pandemic), and hand hygiene or hand washing promotion interventions. A second reviewer provided feedback until consensus was reached (for full search strategies see Additional file 2).

### Study selection

Retrieved references were imported in Endnote, duplicates were removed, and title/abstract screening was performed by one reviewer, before assessing whether full texts met the in−/exclusion criteria. A second reviewer repeated the full text evaluation process and a final set of included studies was compiled upon consensus. Included studies were used to identify additional studies by searching reference lists and their 20 first related citations in MEDLINE (PubMed interface).

### Eligibility criteria

We only included published controlled experimental studies implementing a campaign or program, aimed at the promotion of hand hygiene. These programs consisted of an educational hand hygiene-related component (“software component”: e.g. information leaflets, training or instructions on how or how frequent to practice hand hygiene, …) and optionally provision of materials, including soap, alcohol-based hand rubs or others (“hardware component”). We excluded studies that compared this type of intervention to a control group with an educational component only. Studies combining both facemask and hand hygiene components were also included if the control group had a facemask component only, allowing the evaluation of the additional effect of the hand hygiene component. At the population level we included studies with otherwise healthy people in the community setting (e.g. school, office or household setting), during the COVID-19 pandemic or another pandemic or epidemic of respiratory droplet-transmissible diseases. Primary outcomes represent measures of disease spread including the number of laboratory-confirmed cases (positive for the infectious agent causing the disease) and number of hospital admissions (for patients with laboratory-confirmation of the disease). Secondary outcomes included health outcomes, such as disease-related absenteeism or (self-reported) cases with influenza-like illness (ILI). Additionally, secondary outcomes included behavioral outcomes, including compliance (e.g. self-reported adoption of proper hand washing techniques or product usage as an indirect measurement). During the interepidemic period of an epidemic-prone droplet-transmissible disease (e.g. influenza), only studies reporting laboratory-confirmed measures of infection were included. Studies performed in the (pre-)hospital/health care setting, as well as studies performed during outbreaks of diseases with modes of transmission, other than infectious respiratory droplets, for example through fecal-oral transmission (e.g. cholera) or vector-mediated transmission (e.g. Zika virus disease) were excluded. Only English language studies were included.

### Data extraction

Study characteristics (study type, country of intervention, (inter)epidemic period, population) and study findings were extracted and tabulated by a single reviewer. The country in which the intervention of a study was implemented was classified as either low- to middle-income country (LMIC) or high-income country (HIC), as defined by the World Bank [10]. Additionally, this reviewer also extracted cluster information (cluster size and intracluster correlation coefficient (ICC), used to determine the design effect (DE)), needed for adjustments for clustering effects of cluster-RCTs.

### Data synthesis

Where possible, meta-analyses were performed in Cochrane’s RevMan 5.4 software, using outcomes adjusted for clustering effects according to the Cochrane Handbook, resolving the issue of otherwise artificially narrow confidence intervals [11]. A *p*-value < 0.05 was considered statistically significant.

### Grading of the evidence

Limitations in experimental study designs were analyzed by assessing appropriateness of randomization, allocation concealment and blinding, as well as completeness of accounting of outcome events and selectivity of outcome reporting, as proposed by the Grading of Recommendations Assessment, Development and Evaluation (GRADE) Working Group. This evaluation was followed by a certainty rating of the body of evidence, according to the GRADE methodology [12].

## Results

### Description of studies

#### Study selection

**Fig. 1:**
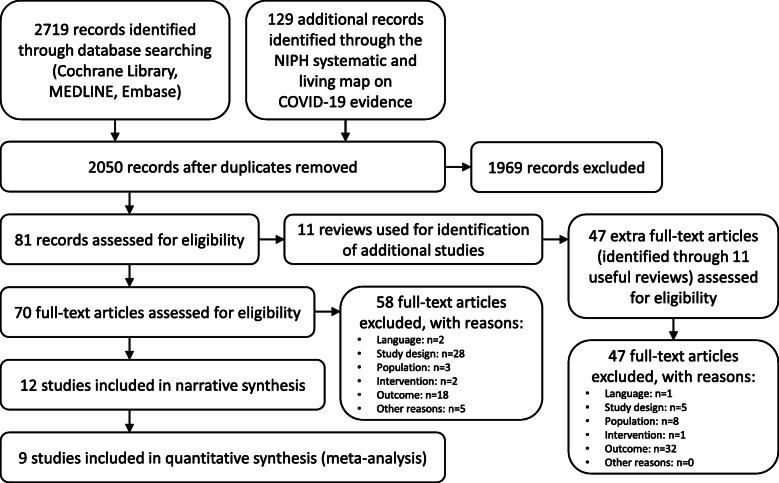
PRISMA flowchart for the selection of eligible studies

Out of 2050 unique references we selected 12 relevant studies (Fig. 1), all of which were cluster-RCTs [13–24]. In addition, 11 systematic reviews, withheld after title/abstract screening, were used to identify extra studies, however, none of these 47 extra studies met the in−/exclusion criteria. Screening of the reference lists of included studies and their 20 first related citations did not yield additional studies.

#### Study characteristics

No studies on the effectiveness of handwashing promotion activities during spread of COVID-19 or respiratory droplet-transmissible diseases other than influenza were withheld. We included 1 study, performed during both an epidemic as well as an interepidemic period of influenza, of which we extracted the data for both periods [23]. In addition, 4 included studies reported study data from an epidemic period [18–21], whereas another 7 included studies reported study data from an interepidemic period [13–17, 22, 24]. The setting of these studies varied from the household setting (*n* = 5) [16, 17, 19, 21, 23], over the university (*n* = 2) [13, 14] and school setting (*n* = 4) [15, 18, 22, 24] to the office setting (*n* = 1) [20]. Of the 12 included studies, 5 studies were performed in LMICs, of which one was conducted in the African continent [24] and 4 others in the Asian continent [15, 18, 19, 21]. The 7 studies in HICs were either performed in the USA [13, 14, 22], Europe [20, 23] or Asia. All 12 studies implemented at least one intervention consisting of a promotional program with both a software and a hardware component. The software component was characterized by education of the target group through either verbally communicated hand hygiene lessons (e.g. instructions by telephone or face-to-face), with training or instructions on how and how frequent to practice hand hygiene [15, 18, 20], or in combination with written or visual media (e.g. information leaflets, posters, video/live demonstration) [13, 14, 16, 17, 19, 21–24]. The hardware component consisted of provision of hand hygiene materials, either soap [19, 21, 24], alcohol-based hand sanitizers [13–15, 18, 22, 23] or a combination of both [16, 17, 20], provided by the researchers to every participating individual [13, 14, 24] or alternatively, to be shared within their cluster or with others in case of provision at a common place (e.g common courtyards or school toilets) [15, 18, 19, 21–23]. A combination of provided materials for personal and shared use was also possible [16, 17, 20]. For one study, parents provided bar soap and a clean towel if they could afford this, otherwise, the school provided materials to the children [24]. As comparison, we included studies with a control group in which participants continued usual practices, or in which an alternative educational program unrelated to hand hygiene promotion was implemented (e.g. smoking cessation education [21] or an educational program with lifestyle and dietary tips [16, 17]). Besides this, we also included 3 studies, comparing an intervention group with provision of both face masks and alcohol-based hand sanitizer versus a control group with provision of face masks only (both groups received information on proper use of provided materials), allowing evaluation of the additional effect of the hand hygiene component [13, 14, 23]. Study characteristics are summarized in Table 1 (with more details provided in Additional file 3). A detailed summary of findings table can be found in Additional file 4 and additional cluster information (cluster size, ICC and DE) in Additional file 5.

**Table 1:** Study characteristics

Author Publication year	Country (LMIC or HIC)	Population	Intervention	Comparisons extracted	Health outcome measures extracted
**Epidemic**					
Pandejpong, 2012 [18]	Thailand (LMIC)	68 classrooms of a kindergarten school in Bangkok with 1441 children randomized	Hardware component: Handgel (each classroom) Software component: Instruction to apply every 60 or 120 min versus maintained school standard (handgel applied before lunch)	HH (1/60′) vs HH (1/120′) vs control	- Number of absence episodes due to physician-confirmed ILI (sick days/present days)
Ram, 2015 [19]	USA (HIC)	60 households with index cases and 427 household contacts susceptible for influenza 377 households with index cases and 3159 household contacts susceptible for ILI	Hardware component: Handwashing station (central location) Software component: Group education on hand washing versus continuation of usual hand hygiene practices	HH vs control	- Influenza positive cases - ILI positive cases
Savolainen-Kopra, 2012 [20]	Finland (HIC)	9 office work units with 325 persons	Hardware component: Liquid hand soap or handgel (in toilets and for personal use) Software component: Instructions and recommendations to limit infection transmission versus continuation of usual hand hygiene practices (liquid hand soap provided at work)	HH_1_ (soap and water) or HH_2_ (alcohol-based) vs control	- Number of reported respiratory infection episodes (per total reported weeks)
Simmerman, 2011 [21]	Thailand (LMIC)	238 households with index cases (children only) 594 household contacts susceptible for influenza	Hardware component: Liquid hand soap (each household) using graduated dispenser system Software component: Individual handwashing education and training versus an educational control group receiving education unrelated to hand hygiene (nutritional, physical activity and smoking cessation education)	HH vs control	- Influenza positive cases - ILI positive cases
Suess, 2012 [23]	Germany (HIC)	28 households with index cases and 70 household contacts susceptible for influenza	Hardware component: Face mask, combined with or without handgel Software component: Information on proper use (written information, instructions by telephone and demonstration)	FM + HH vs FM	- Influenza positive cases - ILI positive cases
**Interepidemic**					
Aiello, 2010 [13]	USA (HIC)	5 residence halls with 745 young adults	Hardware component: Face mask, combined with or without handgel Software component: Information on proper use (educational video on face mask use and hand hygiene, plus written information on hand sanitizer use)	FM + HH vs FM	- Influenza positive cases
Aiello, 2012 [14]	USA (HIC)	25 residence houses with 741 young adults		FM + HH vs FM	- Influenza positive cases
Biswas, 2019 [15]	Bangladesh (LMIC)	24 primary schools with 10,855 students	Hardware component: Handgel (each classroom and in toilets) Software component: Education versus continuation of usual hand hygiene practices	HH vs control	- Influenza positive cases
Cowling, 2008 [17]	Hong Kong (HIC)	101 households with index cases and 289 household contacts	Hardware component: Handgel (automatic sanitizer and individual bottles) and liquid soap (each household) Software component: Education versus an educational control group receiving education unrelated to hand hygiene (about healthy diet and lifestyle)	HH vs control	- Influenza positive cases
Cowling, 2009 [16]	Hong Kong (HIC)	176 households with index cases and 536 household contacts		HH vs control	- Influenza positive cases
Stebbins, 2011 [22]	USA (HIC)	10 elementary schools with 3360 students	Hardware component: Handgel (each classroom and in common school areas) Software component: Hand and respiratory hygiene training and education on influenza versus continuation of usual hand hygiene practices	HH vs control	- Influenza positive cases
Suess, 2012 [23]	Germany (HIC)	26 households with index cases and 66 household contacts susceptible for influenza	Hardware component: Face mask, combined with or without handgel Software component: Information on proper use (written information, instructions by telephone and demonstration)	FM + HH vs FM	- Influenza positive cases
Talaat, 2011 [24]	Egypt (LMIC)	60 elementary schools with 44,451 students	Hardware component: Bar soap provided by school administration (or by parents if they could afford it) Software component: Educational campaign material (posters, booklets, informational flyers, …) and hand hygiene activities versus continuation of usual hand hygiene practices (if done at all)	HH vs control	- Influenza positive cases

*LMIC* low- to middle-income country, *HIC* high-income country, *HH* hand hygiene, *FM* face mask, *ILI* influenza-like illness

### Effect of interventions

#### Epidemic period

**Fig. 2:**
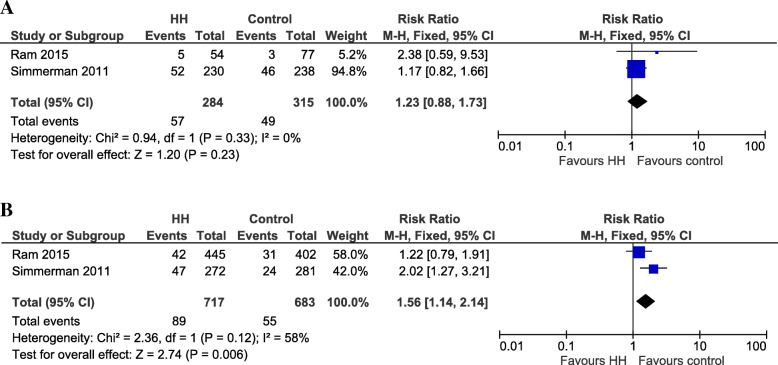
Cases during an epidemic period. Secondary **A** influenza positive and **B** ILI positive cases in household settings for hand hygiene promotion program versus control

Five studies, reporting health outcomes, were performed during an epidemic period of influenza virus [18–21, 23], of which two were on household transmission of influenza and influenza-like illness (ILI) [19, 21]. Typically, in this type of household study setting, the hand hygiene promotion intervention starts upon identification of an index case, i.e. an influenza positive case, as confirmed by laboratory testing. People living in the same household as the index case are considered as susceptible household contacts and their reported health outcomes were extracted, i.e. the secondary attack rate, to evaluate the effects of the hand hygiene promotion intervention. These two studies, performed in LMICs, reported the number of laboratory-confirmed influenza positive cases when implementing a hand hygiene promotion intervention, in which a handwashing station (with water tap and bar soap) was placed at a central location and households were educated in group on proper hand washing [19] or in which liquid hand soap with gradual dispenser was provided to each household, together with individual handwashing education and training [21]. Both studies compared the intervention to a control group in which household members continued their usual handwashing practices and when meta-analyzed, adjusting for clustering effects, the RR: 1.23, 95%CI [0.88;1.73], (*p* = 0.23) could not demonstrate that susceptible household contacts in the hand hygiene promotion intervention had a lower number of secondary influenza positive cases, compared to the control group (Fig. 2a).

In addition, these studies also reported the number of influenza-like illness (ILI) positive cases and the meta-analysis, adjusted for clustering effects, could not show that the susceptible household contacts in the hand hygiene promotion intervention had a lower number of secondary ILI positive cases, compared to the control group, since the RR: 1.56, 95%CI [1.14;2.14], (*p* = 0.01) was unexpectedly found to be in favor of the control condition (Fig. 2b).

Another study in a household setting [23] reported the number of secondary influenza positive cases in a HIC during the epidemic period, but compared a combined facemask plus hand hygiene promotion intervention to a facemask only control group. Only the combined intervention was provided with alcohol-based hand rub, whereas both groups were provided with face masks and information on the proper use of provided materials, through instructions by telephone, written information and demonstration by personnel. In this study, an additional effect of the hand hygiene component on secondary influenza positive cases could not be demonstrated: RR: 0.78, 95%CI [0.12;5.07], (*p* = 0.79). As for the additional effect of the hand hygiene component on secondary ILI positive cases, a statistically significant difference could not be demonstrated: RR: 0.52, 95%CI [0.10;2.83], (*p* = 0.45), even though more people in the combined facemask plus hand hygiene promotion intervention, compared to the facemask only control group, reported that they disinfected their hands after coming home or after touching objects, RR: 1.83, 95%CI [1.17;2.86], (*p* = 0.01). A study in Thailand [18] compared whether instructing children in kindergarten to apply alcohol-based sanitizer either every hour or every 2 hours differentially affected absenteeism rates, because of physician-confirmed ILI, when compared to control (i.e. maintaining current school standards, applying it once before lunch). A statistically significant effect was only found for the rate of absent to present days when hand sanitizer was applied every 60 min, RD: 0.01, 95%CI [0.00;0.02], (*p* = 0.00), but not when applied every 120 min, RD: 0.00, 95%CI [− 0.01;0.01], (*p* = 0.74), compared to control; data were not adjusted for the clustering effect. Lastly, a study in a HIC office setting, in which either liquid hand soap or alcohol-based hand rub was provided in toilets at work and for personal use, reported the number of respiratory illness episodes per total reported weeks [20]. Interventions were accompanied by guidance and recommendations regarding hand hygiene and limiting infection transmission, not provided to the control group [20]. This study could not show an effect in favor of its intervention, RR: 1.04, 95%CI [0.92;1.17], (*p* = 0.52) (soap and water) and RR: 1.13, 95%CI [1.00;1.28], (*p* = 0.04) (alcohol-based hand rub) [20], but data were not adjusted for the clustering effect.

#### Interepidemic period

**Fig. 3:**
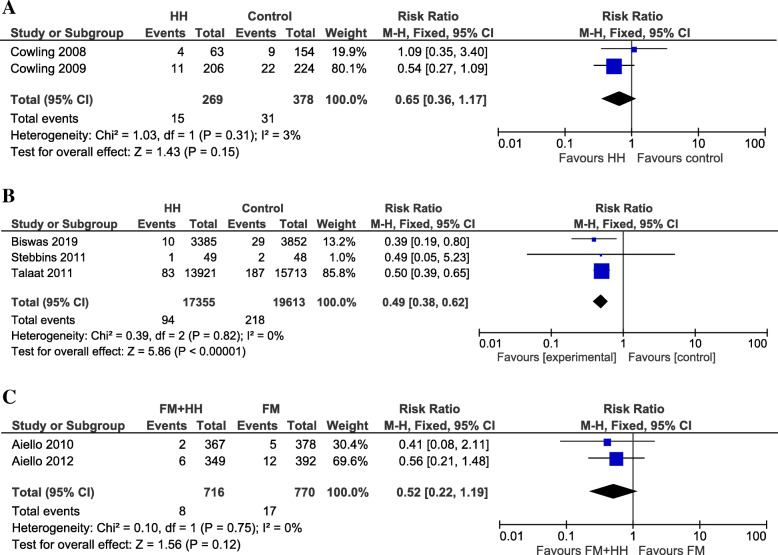
Cases during an interepidemic period. **A** Secondary influenza positive cases in household settings and **B** influenza positive cases in school settings for hand hygiene promotion program versus control and **C** influenza positive cases in university settings for hand hygiene promotion plus facemask program versus facemask program only

We also included studies performed during an interepidemic period of epidemic-prone diseases (e.g. influenza). We used two studies conducted in a household setting with index cases [16, 17] for a clustering effect-adjusted meta-analysis. The underpowered pilot study [17] published in 2008 and its follow-up study in 2009 [16] were performed in a HIC (Hong Kong) and implemented an educational program (about healthy diets and lifestyle), unrelated to hand hygiene, for both intervention and control group. The intervention group additionally received hand hygiene education, including hand washing demonstration, and was provided with an automatic alcohol-based hand sanitizer, liquid hand soap for each household and individual small bottles of alcohol-based handgel. The meta-analysis of these studies could not demonstrate a beneficial effect for a hand hygiene promotion program on the number of secondary laboratory-confirmed influenza cases compared to the control group, RR: 0.65, 95%CI [0.36;1.17], (*p* = 0.15) (Fig. 3a).

A second meta-analysis included 3 studies performed in school settings [15, 22, 24] and revealed that the hand hygiene promotion program had a beneficial effect, since the number of laboratory-confirmed influenza cases was lower in hand hygiene promotion intervention, compared to the control group, RR: 0.49, 95%CI [0.38;0.62], (*p* < 0.0001) (Fig. 3b). Two of these studies provided alcohol-based hand sanitizer to each classroom and in common areas, plus hand hygiene education for the LMIC study [15] or plus hand and respiratory hygiene training and influenza education for the HIC study [22]. In the third study, schoolchildren receiving the hand hygiene promotion program were provided with bar soap by the school, if their parents could not afford it (of note, the study was performed in a LMIC), and received an educational hand hygiene campaign (with hand hygiene activities and written and visual information materials) [24].

Lastly, we evaluated the additional effect of the hand hygiene component in 3 studies [13, 14, 23] comparing the number of (secondary) influenza positive cases in the group receiving a facemask plus hand hygiene program to the group receiving a facemask program only. Besides provision of face masks with or without handgel, interventions also included information on their proper use, either through an educational video (on face mask use and hand hygiene) combined with written information on hand sanitizer use [13, 14], or through instructions by telephone, written information and demonstration by personnel [23]. An additional effect of the hand hygiene component on the number of secondary influenza positive cases could not be demonstrated in the study performed in a household setting with index cases, RR: 3.42, 95%CI [0.74;15.79], (*p* = 0.12) [23]. Of note, measures for self-reported hand disinfection were not significantly different between these two groups. Also, the meta-analysis of the two studies conducted in a university setting could not demonstrate an additional effect of the hand hygiene component on influenza positive cases, RR: 0.52, 95%CI [0.22;1.19], (*p* = 0.12) [13, 14] (Fig. 3c).

### Risk of bias in included studies

**Fig. 4:**
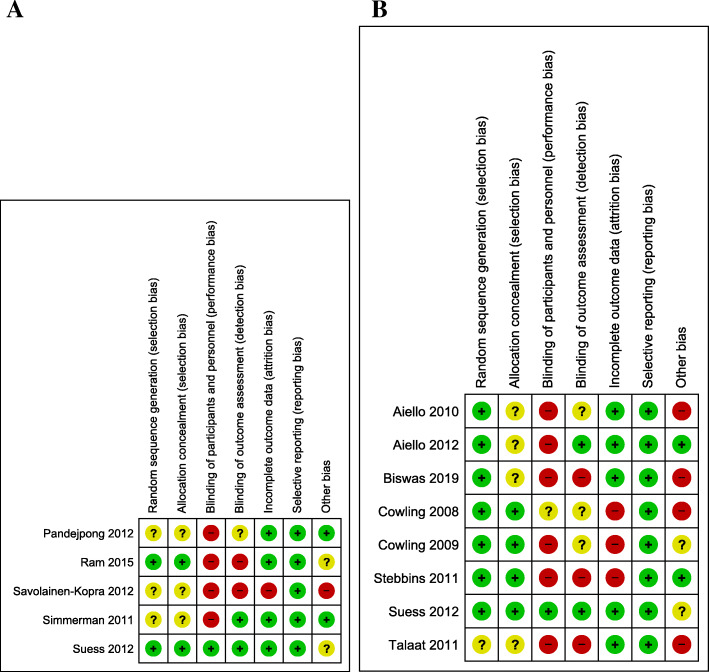
Risk of bias assessment. Assessment for studies performed during **A** an epidemic period and **B** an interepidemic period

Finally, we assessed and summarized the limitations in study design for all included studies individually in Additional file 6. All 12 included studies were cluster-RCT studies, leading to an initial high certainty level. The certainty of evidence was downgraded by −2 for studies performed during an epidemic period, due to risk of bias (Fig. 4a) and imprecision of results, resulting in low certainty evidence. For studies during an interepidemic period, the certainty of evidence was downgraded by −2, due to risk of bias (Fig. 4b) and imprecision of results, resulting in low certainty evidence, except for the comparison where a hand hygiene promotion intervention in the school setting was compared to control and in which we downgraded due to risk of bias by −1, which resulted in moderate certainty evidence, since there was no imprecision of results.

## Discussion

In this rapid review, we identified 12 cluster-RCTs, assessing the effect of hand hygiene promotion programs during epidemics of respiratory droplet-transmissible infections on health outcomes and behavioral outcomes in otherwise healthy people in the community setting. All studies were conducted in the context of influenza infections. We did not find controlled experimental studies in the context of COVID-19, assessing health outcomes upon the promotion of hand hygiene interventions. Those studies conducted during the COVID-19 pandemic focused on hand hygiene as such (e.g. using different types of hand sanitizer) instead of the promotion of hand hygiene, or were performed in a hospital setting instead of the community setting. We also encountered observational studies (e.g. cross-sectional studies), which did not meet our experimental study design inclusion criterium. The fact that no controlled studies are available yet is not surprising, since setting up a rigorous controlled study takes time. We did, however, find one interesting record, describing a protocol for a randomized controlled trial to promote proper COVID-19 hand hygiene practices, using a short, animated video [25].

The studies we identified were performed during epidemic periods of influenza, however, the effect on influenza transmission (secondary attack rate) could not be demonstrated in LMICs implementing educational handwashing interventions in households where one household member was already infected [19, 21]. Similar results were found in a meta-analysis of 2 studies in households from interepidemic periods [16, 17]. Importantly, this type of intervention is reactive in nature: the intervention is initiated after household index cases are identified. This contrasts with interventions in other settings, e.g. school settings, which are implemented regardless of case identification, and thus are more preventive in nature. School absenteeism in an epidemic period was only reduced upon hand hygiene promotion when hands were washed very frequently [18] (unadjusted data). For the meta-analysis of 3 studies performed in schools during interepidemic periods, the number of influenza positive cases was lower in the group receiving the hand hygiene promotion intervention and the evidence was of moderate certainty [15, 22, 24]. At the office, beneficial effects on respiratory illness could not be demonstrated, but only unadjusted data from one study in an epidemic period were available [20]. Also, regardless of (inter)epidemic period or setting, the effect of an additional handwashing intervention to facemask intervention on influenza or ILI transmission could not be demonstrated [13, 14, 23].

We believe that there are several plausible reasons for the lack of an overall effect of hand hygiene promotion interventions in different settings and periods (epidemic vs interepidemic). For studies performed in the household setting, handwashing promotion is probably more effective when applied soon after illness onset of index patients. Transmission within households might already have occurred when delaying interventions or human behavior might not change rapidly enough to curb transmission. Also, specific home arrangements can interfere with hand hygiene promotion effectiveness, e.g. homes in LMICs can be more crowded and ventilation limited, especially when children and parents share sleeping arrangements [26], allowing much faster (aerosolized) viral transmission. In some cases, it is possible that children are most susceptible and contagious for a certain infection. It is therefore crucial that programs are tailored to the appropriate developmental stage, since their effectiveness will be limited if handwashing behavior is only increased in older individuals. Another aspect to take into account when interpreting the study results is the fact that, specifically during epidemic periods, national infection prevention programs are possibly also implemented. Unexpected beneficial effects in control groups, originally designed to refrain from any intervention, could thereby level out the potential effect of the intervention. Of note, there is large heterogeneity in the educational component of the included studies in this review. This ranges from a simple instruction of when to wash hands to combinations of face-to-face instructions, training, live demonstrations and written cues on posters or information leaflets. It seems obvious that the communication strategy and degree of comprehensiveness are determining for hand hygiene promotion effectiveness.

The meta-analysis of two LMIC household studies (epidemic period) unexpectedly reported less ILI cases in favor of the control group, compared to hand hygiene promotion [19, 21]. We do not exclude the possibility that hand hygiene materials (e.g. hand washing stations or actuators of alcohol-based hand sanitizers) serve as a fomite for pathogen transmission. It is also plausible that the intervention increased the potential for transmission, for example through increasing the number of social contacts within intervention group participants, when simultaneously gathering around hand hygiene stations (e.g. before lunch).

This systematic review has several limitations. Firstly, because of time pressure, most steps of the review process were done by only one reviewer; one additional reviewer only checked the search strategy and independently evaluated studies at the full text level. Secondly, the number of screened databases was limited to three main databases and the NIPH systematic and living map on COVID-19 evidence. However, we did try to cover this by using sensitive search strategies and by screening all relevant systematic reviews for potentially interesting studies – but did not identify additional studies to include.

For future studies in epidemics of respiratory droplet-transmissible diseases to come, this systematic review can be useful, since it highlights the need to implement hand hygiene promotion programs early on, ideally before infection occurs. Also, the measurement of hand washing behaviour (whether or how many times hands are sanitized) is crucial, since these data allow to explain a possible lack of hand hygiene promotion effectiveness, by the nature of an ineffective intervention or by the lack of adherence to an effective intervention. Additionally, reporting sociocultural factors that influence hand hygiene practices could explain why the effect of certain hand hygiene promotion interventions could not be shown. It would also be useful to report the contribution of governmental public health interventions; even more, evaluation of promotional campaigns using RCTs is not always warranted when public health interventions are omnipresent. In this systematic review, the meta-analyses were performed on data adjusted for clustering effects (where possible), thereby avoiding artificially narrow confidence intervals. Previous systematic reviews evaluating the effects of hand hygiene promotion programs on laboratory-confirmed influenza performed meta-analyses of cluster-RCTs as if these trials were individual-RCTs, thereby not accounting for clustering effects [27, 28]. We suggest that future studies take clustering effects into account.

## Conclusions

In summary, the findings of our systematic review suggest that when reactive measures are taken, after cases have been identified, the effectiveness of hand hygiene promotion could not be demonstrated, whereas hand hygiene promotion can have beneficial effects on health outcomes when the intervention is implemented as a preventive measure, regardless of whether cases have been identified.

## Supplementary Information

**Additional file 1.** PRISMA checklist.

**Additional file 2.** Search strategies.

**Additional file 3.** Detailed study characteristics.

**Additional file 4.** Summary of findings.

**Additional file 5.** Cluster information.

**Additional file 6.** Risk of bias assessment and summary.

## Data Availability

All data generated or analysed during this study are included in this published article and its supplementary information files.

## Abbreviations

COVID-19: Coronavirus Disease 2019
DE: Design effect
FM: Face mask
GRADE: Grading of Recommendations Assessment, Development and Evaluation
HH: Hand hygiene
HIC: High-income country
ICC: Intracluster correlation coefficient
ILI: Influenza-like illness
LMIC: Low- to middle-income country
MERS: Middle East Respiratory Syndrome
RD: Rate difference
RR: Relative risk
SARS: Severe Acute Respiratory Syndrome
