# Supplementary material for: The effect of hand hygiene promotion programs during epidemics and pandemics of respiratory droplet-transmissible infections on health outcomes: a rapid systematic review
Source: BMC Public Health. 2021 Sep 25;21:1745. doi: 10.1186/s12889-021-11815-4 (PMC8467175; doi:10.1186/s12889-021-11815-4)
Supplement: Supplementary file 2 — Additional file 2. Search strategies. [file 12889_2021_11815_MOESM2_ESM.docx]

**Additional file 2: Search strategies**

**Cochrane Library**

[mh ”SARS Virus”] OR “Severe Acute Respiratory Syndrome”:ti,ab,kw OR SARS:ti,ab,kw OR “SARS-COV”:ti,ab,kw OR [mh “Middle East Respiratory Syndrome Coronavirus”] OR “Middle East Respiratory Syndrome Coronavirus”:ti,ab,kw OR MERS:ti,ab,kw OR [mh “Coronavirus Infections”] OR “coronavirus infection”:ti,ab,kw OR “coronavirus infections”:ti,ab,kw OR “corona virus”:ti,ab,kw OR coronavirus:ti,ab,kw OR “corona viruses”:ti,ab,kw OR coronaviruses:ti,ab,kw OR “SARS-COV2”:ti,ab,kw OR “SARS-COV-2”:ti,ab,kw OR “covid-19”:ti,ab,kw OR “COVID19”:ti,ab,kw OR [mh ”Influenza, Human”] OR influenza:ti,ab,kw OR flu:ti,ab,kw OR orthomyxovirus:ti,ab,kw **OR** ([mh “Epidemics”] OR epidemic:ti,ab,kw OR epidemics:ti,ab,kw OR outbreak:ti,ab,kw OR outbreaks:ti,ab,kw OR pandemic:ti,ab,kw OR pandemics:ti,ab,kw OR “public health emergency”:ti,ab,kw OR [mh ^“Disease Outbreaks”]) **AND** ([mh ^”Respiratory Tract Infections”] OR “respiratory tract infection”:ti,ab,kw OR “respiratory tract infections”:ti,ab,kw OR “respiratory infection”:ti,ab,kw OR “respiratory infections”:ti,ab,kw OR “respiratory virus”:ti,ab,kw OR “respiratory viruses”:ti,ab,kw OR “respiratory illness”:ti,ab,kw OR “respiratory illnesses”:ti,ab,kw OR “pneumococcal disease”:ti,ab,kw OR pneumococcus:ti,ab,kw OR “streptococcus pneumoniae”:ti,ab,kw OR [mh “Tuberculosis, Pulmonary”] OR tuberculosis:ti,ab,kw OR meningitis:ti,ab,kw OR “foot-mouth-disease”:ti,ab,kw OR “foot-and-mouth-disease”:ti,ab,kw OR polio:ti,ab,kw OR poliomyelitis:ti,ab,kw OR poliovirus:ti,ab,kw OR [mh “Diphteria”] OR diphteria:ti,ab,kw OR diphteriae:ti,ab,kw OR [mh “Plague”] OR plague:ti,ab,kw OR pestis:ti,ab,kw OR [mh ”Paramyxoviridae Infections”] OR paramyxoviridae:ti,ab,kw OR paramyxovirus:ti,ab,kw OR [mh “Respiratory Syncytial Virus, Human”] OR “respiratory syncytial virus”:ti,ab,kw OR RSV:ti,ab,kw OR pneumovirus:ti,ab,kw OR [mh “Nipah Virus”] OR “nipah virus”:ti,ab,kw OR henipavirus:ti,ab,kw OR mumps:ti,ab,kw OR rubulavirus:ti,ab,kw OR measles:ti,ab,kw OR rubeola:ti,ab,kw OR morbillivirus:ti,ab,kw OR [mh ”Variola virus”] OR variola:ti,ab,kw OR smallpox:ti,ab,kw OR monkeypox:ti,ab,kw)

AND

[mh “Hand Hygiene”] OR “hand hygiene”:ti,ab,kw OR handhygiene:ti,ab,kw OR “hand washing”:ti,ab,kw OR handwashing:ti,ab,kw OR “hand wash”:ti,ab,kw OR handwash:ti,ab,kw OR [mh “Hand Sanitizers”] OR “hand sanitizers”:ti,ab,kw OR handsanitizers:ti,ab,kw OR “hand sanitizer”:ti,ab,kw OR handsanitizer:ti,ab,kw OR “hand sanitization”:ti,ab,kw OR “handsanitization”:ti,ab,kw OR “hand rub”:ti,ab,kw OR handrub:ti,ab,kw OR “hand cleaning”:ti,ab,kw OR handcleaning:ti,ab,kw OR “hand cleaner”:ti,ab,kw OR handcleaner:ti,ab,kw OR “hand cleansing”:ti,ab,kw OR handcleansing:ti,ab,kw OR “hand cleanser”:ti,ab,kw OR handcleanser:ti,ab,kw OR “hand disinfection”:ti,ab,kw

**MEDLINE (via the PubMed interface)**

“SARS Virus”[Mesh] OR “Severe Acute Respiratory Syndrome”[TIAB] OR SARS[TIAB] OR “SARS-COV”[TIAB] OR “Middle East Respiratory Syndrome Coronavirus”[Mesh] OR “Middle East Respiratory Syndrome Coronavirus”[TIAB] OR MERS[TIAB] OR "Coronavirus Infections"[Mesh] OR “coronavirus infection”[TIAB] OR “coronavirus infections”[TIAB] OR “corona virus”[TIAB] OR coronavirus[TIAB] OR “corona viruses”[TIAB] OR coronaviruses[TIAB] OR “SARS-COV2”[TIAB] OR “SARS-COV-2”[TIAB] OR “covid-19”[TIAB] OR “COVID19”[TIAB] OR “Influenza, Human”[Mesh] OR influenza[TIAB] OR flu[TIAB] OR orthomyxovirus[TIAB] **OR** (“Epidemics”[Mesh] OR epidemic[TIAB] OR epidemics[TIAB] OR outbreak[TIAB] OR outbreaks[TIAB] OR pandemic[TIAB] OR pandemics[TIAB] OR “public health emergency”[TIAB] OR “Disease Outbreaks”[Mesh:NoExp]) **AND** (“Respiratory Tract Infections”[Mesh:NoExp] OR “respiratory tract infection”[TIAB] OR “respiratory tract infections”[TIAB] OR “respiratory infection”[TIAB] OR “respiratory infections”[TIAB] OR “respiratory virus”[TIAB] OR “respiratory viruses”[TIAB] OR “respiratory illness”[TIAB] OR “respiratory illnesses”[TIAB] OR “pneumococcal disease”[TIAB] OR pneumococcus[TIAB] OR “streptococcus pneumoniae”[TIAB] OR “Tuberculosis, Pulmonary”[Mesh] OR tuberculosis[TIAB] OR meningitis[TIAB] OR “foot-mouth disease”[TIAB] OR “foot-and-mouth disease”[TIAB] OR polio[TIAB] OR poliomyelitis[TIAB] OR poliovirus[TIAB] OR “Diphteria”[Mesh] OR diphteria[TIAB] OR diphteriae[TIAB] OR “Plague”[Mesh] OR plague[TIAB] OR pestis[TIAB] OR “Paramyxoviridae Infections”[Mesh] OR paramyxoviridae[TIAB] OR paramyxovirus[TIAB] OR “Respiratory Syncytial Virus, Human”[Mesh] OR “respiratory syncytial virus”[TIAB] OR RSV[TIAB] OR pneumovirus[TIAB] OR “Nipah Virus”[Mesh] OR “nipah virus”[TIAB] OR henipavirus[TIAB] OR mumps[TIAB] OR rubulavirus[TIAB] OR measles[TIAB] OR rubeola[TIAB] OR morbillivirus[TIAB] OR “Variola virus”[Mesh] OR variola[TIAB] OR smallpox[TIAB] OR monkeypox[TIAB])

AND

“Hand Hygiene”[Mesh] OR “hand hygiene”[TIAB] OR handhygiene[TIAB] OR “hand washing”[TIAB] OR handwashing[TIAB] OR “hand wash”[TIAB] OR handwash[TIAB] OR “Hand Sanitizers”[Mesh] OR “hand sanitizers”[TIAB] OR handsanitizers[TIAB] OR “hand sanitizer”[TIAB] OR handsanitizer[TIAB] OR “hand sanitization”[TIAB] OR “handsanitization”[TIAB] OR “hand rub”[TIAB] OR handrub[TIAB] OR “hand cleaning”[TIAB] OR handcleaning[TIAB] OR “hand cleaner”[TIAB] OR handcleaner[TIAB] OR “hand cleansing”[TIAB] OR handcleansing[TIAB] OR “hand cleanser”[TIAB] OR handcleanser[TIAB] OR “hand disinfection”[TIAB]

**Embase (via the Embase.com interface)**

‘SARS coronavirus’/exp OR ‘Severe Acute Respiratory Syndrome’:ab,ti OR SARS:ab,ti OR ‘SARS-COV’:ab,ti OR ‘Middle East respiratory syndrome coronavirus’/exp OR ‘Middle East Respiratory Syndrome Coronavirus’:ab,ti OR MERS:ab,ti OR ‘Coronavirus infection’/exp OR ‘coronavirus infection’:ab,ti OR ‘coronavirus infections’:ab,ti OR ‘corona virus’:ab,ti OR coronavirus:ab,ti OR ‘corona viruses’:ab,ti OR coronaviruses:ab,ti OR ‘SARS-COV2’:ab,ti OR ‘SARS-COV-2’:ab,ti OR ‘covid-19’:ab,ti OR ‘COVID19’:ab,ti OR ‘influenza’/exp OR influenza:ab,ti OR flu:ab,ti OR orthomyxovirus:ab,ti **OR** (‘epidemic’/exp OR epidemic:ab,ti OR epidemics:ab,ti OR outbreak:ab,ti OR outbreaks:ab,ti OR ‘pandemic’/exp OR pandemic:ab,ti OR pandemics:ab,ti OR ‘public health emergency’:ab,ti) **AND** (‘respiratory tract infection’/de OR ‘respiratory tract infection’:ab,ti OR ‘respiratory tract infections’:ab,ti OR ‘respiratory infection’:ab,ti OR ‘respiratory infections’:ab,ti OR ‘respiratory virus’:ab,ti OR ‘respiratory viruses’:ab,ti OR ‘respiratory illness’:ab,ti OR ‘respiratory illnesses’:ab,ti OR ‘pneumococcal disease’:ab,ti OR pneumococcus:ab,ti OR ‘streptococcus pneumoniae’:ab,ti OR ‘tuberculosis’/exp OR tuberculosis:ab,ti OR meningitis:ab,ti OR ‘foot-mouth disease’:ab,ti OR ‘foot-and-mouth disease’:ab,ti OR polio:ab,ti OR poliomyelitis:ab,ti OR poliovirus:ab,ti OR ‘diphteria’/exp OR diphteria:ab,ti OR diphteriae:ab,ti OR ‘plague’/exp OR plague:ab,ti OR pestis:ab,ti OR ‘paramyxovirus infection’/exp OR paramyxoviridae:ab,ti OR paramyxovirus:ab,ti OR ‘Human respiratory syncytial virus’/exp OR ‘respiratory syncytial virus’:ab,ti OR RSV:ab,ti OR pneumovirus:ab,ti OR ‘Nipah virus’/exp OR ‘nipah virus’:ab,ti OR henipavirus:ab,ti OR mumps:ab,ti OR rubulavirus:ab,ti OR measles:ab,ti OR rubeola:ab,ti OR morbillivirus:ab,ti OR ‘Smallpox virus’/exp OR ‘smallpox’/exp OR variola:ab,ti OR smallpox:ab,ti OR monkeypox:ab,ti)

AND

‘hand washing’/exp OR ‘hand hygiene’:ab,ti OR handhygiene:ab,ti OR ‘hand washing’:ab,ti OR handwashing:ab,ti OR ‘hand wash’:ab,ti OR handwash:ab,ti OR ‘hand sanitizer’/exp OR ‘hand sanitizers’:ab,ti OR handsanitizers:ab,ti OR ‘hand sanitizer’:ab,ti OR handsanitizer:ab,ti OR ‘hand sanitization’:ab,ti OR ‘handsanitization’:ab,ti OR ‘hand rub’:ab,ti OR handrub:ab,ti OR ‘hand cleaning’:ab,ti OR handcleaning:ab,ti OR ‘hand cleaner’:ab,ti OR handcleaner:ab,ti OR ‘hand cleansing’:ab,ti OR handcleansing:ab,ti OR ‘hand cleanser’:ab,ti OR handcleanser:ab,ti OR ‘hand disinfection’:ab,ti

**NIPH systematic and living map on COVID-19 evidence (**[**EPPI-Mapper (nornesk.no)**](https://www.nornesk.no/forskningskart/NIPH_mainMap.html)**)**

Search terms:

- handwashing
- hand washing
- hand hygiene
- hand sanitizer
- hand gel

Filter: “Infection prevention and control”
