# Supplementary material for: The effect of hand hygiene promotion programs during epidemics and pandemics of respiratory droplet-transmissible infections on health outcomes: a rapid systematic review
Source: BMC Public Health. 2021 Sep 25;21:1745. doi: 10.1186/s12889-021-11815-4 (PMC8467175; doi:10.1186/s12889-021-11815-4)
Supplement: Supplementary file 3 — Additional file 3. Detailed study characteristics. [file 12889_2021_11815_MOESM3_ESM.docx]

**Additional file 3: Detailed study characteristics**

| **Author**  **Publication year** | **Country of intervention**  **(LMIC or HIC)** | **Epidemic or inter-epidemic** | **Study design** | **Population** | **Intervention** | **Outcome measures** |
| --- | --- | --- | --- | --- | --- | --- |
| **Epidemic** | | | | | | |
| Pandejpong, 2012 | Thailand  (LMIC) | Epidemic | Experimental: Cluster-randomized controlled trial. | 68 classrooms from a large private kindergarten school in Bangkok (with 1441 children) were randomized to one of 3 groups with different frequencies of hand gel application, either the ‘every 60 min’ group (452 children, mean age 50.58 months, 211 males, 241 females), ‘every 120 min’ group (447 children, mean age 51.37 months, 255 males, 192 females) or the control group (540 children, mean age 49.85 months, 335 males, 205 females). Data were collected from December 2009 to February 2010 (the peak season for ILI in Thailand) during a 12-week study. | Intervention 1:  Provision of alcohol-based hand gel to each classroom and instruction by the teacher to apply it every 60 minutes, with monitoring of teachers and research assistants every disinfection round.  Intervention 2:  Provision of alcohol-based hand gel to each classroom and instruction by the teacher to apply it every 120 minutes, with monitoring of teachers and research assistants every disinfection round.  Control:  Provision of alcohol-based hand gel to each classroom and instruction to maintain the current school standard: to apply it once before lunch, with monitoring of teachers and research assistants every disinfection round. | Data were collected for absenteeism caused by physician-confirmed ILI.  [Data for absenteeism caused by total reported ILI (i.e. with and without physician-confirmation) were not extracted.]  [Handwashing behavior was not reported.] |
| Ram, 2015 | Bangladesh  (LMIC) | Epidemic | Experimental: Cluster-randomized controlled trial. | 377 households with ILI positive index cases completed the study that was conducted in 2009 and 2010 in Kishoregonj, a rural area in Bangladesh. Randomization was done for allocation to either the hand hygiene program group (193 households with 1661 household contacts susceptible for ILI, mean age 24.9 years (SD: 19.2), 781 males, 880 females and 24 households with 177 household contacts susceptible for influenza) or the control group (184 households with 1498 household contacts susceptible for ILI, mean age 25.7 years (SD: 19.6), 694 males, 804 females and 36 households with 250 household contacts susceptible for influenza).  Index case-patients were either defined as ILI positive or alternatively as ILI and influenza positive. | Intervention:  Hand hygiene program:  Didactic and interactive group-level education and skills training on hand washing, with behaviour change communication (based on constructs  of Social Cognitive Theory and the Health Belief Model). A handwashing station (water tap and bar soap) was provided at a central location and cue cards in a common area in the courtyard.  Control:  Usual hand hygiene practices continuation. No handwashing stations were provided. | ILI case definition:  For persons ≥5 years old, ILI was defined as history of fever with either cough or sore throat. For persons <5 years old, ILI was defined as fever. (Criteria in 2010 were more restrictive, with index case-patient symptom onset within 48 hours preceding enrollment.)  Households were visited each day by a data collector until the 10^th^ day following the resolution of the index case-patient’s symptoms.  Oropharyngeal swabs were obtained from index cases and household contacts with ILI for laboratory PCR testing for influenza.  [Handwashing behavior was not extracted, since this outcome was only measured in the intervention group and could therefore not be compared to the control group.]  ICC: In the intention-to-treat analysis for ILI for the calculation of the SAR (secondary attack rate) ratio, the intracluster correlation was 0.37 (Ram 2015).  ICC = 0.37 was used for the adjusted cluster-RCT analysis (this review). |
| Savolainen-Kopra, 2012 | Finland  (HIC) | Epidemic | Experimental: Cluster-randomized controlled trial. | 21 office work units (683 persons) in six corporations in the Helsinki Region were enrolled in the study (with a 16 month intervention period, including the emergence of the 2009 influenza pandemic: data collection between November 2008 and May 2010) and randomized to one of three study arms: 257 persons were allocated to the hand hygiene program with soap and water (mean age 45.1 (range 22-64)), 202 persons were in the hand hygiene program with alcohol-based hand rub (mean age 42.7 (range 20-63)) and 224 persons were in the control group (mean age 42.8 (range 21-62)). For the epidemic period (after end of July 2009 to the end of May 2010), there were 3 offices work units with 109 persons in the hand hygiene program with soap and water, 3 offices work units with 97 persons in the hand hygiene program with alcohol-based hand rub and 3 offices work units with 119 persons in the control group.  [Gender distribution not reported.] | Intervention 1:  Hand hygiene program with soap and water:  Standardized instructions on how to limit infection transmission together with behavioural recommendations. Liquid hand soap was provided in toilets at work and for personal use.  Intervention 2:  Hand hygiene program with alcohol-based hand rub:  Standardized instructions on how to limit infection transmission together with behavioural recommendations. Alcohol-based hand rub was provided in toilets at work and for personal use.  Control:  Usual hand hygiene practices continuation with liquid hand soap provided in toilets at work.  Did not receive any guidance regarding hand hygiene or limiting transmission of infections. | Infection symptoms (respiratory and gastrointestinal infections) and information about sick leave and absences from work were collected through weekly self-reported internet-based questionnaires.  The paper analysed the epidemic period (after the end of July 2009 to the end of May 2010) separately from the first interepidemic period (before the end of July 2009).  [Self-recorded use of soap and alcohol-based disinfectant for personal use was not extracted, since this outcome was only measured in the intervention groups and could therefore not be compared to the control group.]  [Gastrointestinal infection episodes were not extracted.]  [The first period (interepidemic period) was not extracted since no laboratory-confirmed health outcomes were reported.] |
| Simmerman, 2011 | Thailand  (LMIC) | Epidemic | Experimental: Cluster-randomized controlled trial. | 465 households with an influenza positive index case (aged 1 month to 15 years) were enrolled for the study that was conducted between April 2008 and August 2009 (including the first wave of the 2009 influenza A/H1N1 pandemic in June 2009). Randomization was done at a 1:1:1 ratio over the hand hygiene program group (in which 119 index cases (60 males, 59 females) with 292 household contacts (117 males, 175 females) were included for analysis), hand hygiene plus face mask program group (in which 110 index cases (63 males, 47 females) with 291 household contacts (119 males, 172 females) were included for analysis) and the control group (in which 119 index cases (69 males, 50 females) with 302 household contacts (126 males, 176 females) were included for analysis). The median age of index cases was 5.5 years and the median age of household contacts was 34 years.  Index cases were defined as children aged 1 month through 15 years, residents of the Bangkok metropolitan area, with onset of illness <48 hours before respiratory specimens tested positive for influenza by a rapid influenza diagnostic test (that was later confirmed by PCR). | Intervention:  Hand hygiene program:  Hand-washing education was given through interactive and individual hand-washing training with messages about why, when and how to wash (following Thailand Ministry of Public Health guidelines). A hand-washing kit, including graduated dispenser with standard unscented liquid hand soap, was provided to each household.  Control:  Received nutritional, physical activity, and smoking cessation education.  Extra intervention:  Hand hygiene plus face mask program:  Similarly received the hand washing education and hand washing kit and additionally, a box of 50 standard paper surgical face masks and 20 pediatric face masks.  [Data for the hand hygiene plus face mask program group program were not extracted.] | Secondary cases were defined as household members with influenza (defined as a positive PCR result on days 3 or 7 or a fourfold rise in hemagglutinin inhibition antibody titers, with the virus type and subtype matching the index case.)  Households received 4 visits during the observation period of 21 days. Nasal and throat swab specimens were obtained on days 0⁄1, 3, and 7 from the index case and all household members (and tested for influenza by PCR).  (Blood specimens were collected from each consenting household member on day 0⁄1 and again on day 21 for serological testing – hemagglutinin inhibition assay to indirectly assess acute influenza infection.)  ILI case definition: For children <2 years of age, ILI was defined as fever (>38°C) and one or more of the following symptoms; nasal discharge ⁄ congestion, cough, conjunctivitis, respiratory distress (tachypnea, retractions), sore throat, and new seizure. For children aged ≥2 years, ILI was defined as fever and cough or sore throat in the absence of another explanation.  ICC: For PCR or serology outcomes to compare across the three intervention arms, a within-household correlation of 0.18 was used, whereas for ILI outcomes 0.05 was used (Simmerman 2011).  ICC = 0.18 was used for the adjusted cluster-RCT analysis for the influenza outcome (this review).  ICC = 0.05 was used for the adjusted cluster-RCT analysis for the ILI outcome (this review). |
| Suess, 2012 | Germany  (HIC) | Epidemic and interepidemic | Experimental: Cluster-randomized controlled trial. | 84 households with an influenza positive index case (28 in the face mask plus hand hygiene group, 26 in the face mask group and 30 in the control group) and 218 household contacts (67 household contacts in the face mask plus hand hygiene group, 69 in the face mask group and 82 in the usual practices continuation group) were recruited for the study that was conducted during two consecutive influenza seasons (the pandemic season November 2009-January 2010 and January-April 2011) in Berlin, Germany.  In the 2009/2010 season there were 17 index cases (median age 7 years (IQR: 4-10); 10 males, 7 females) and 39 household contacts (median age 34 years (IQR: 19-42); 17 males, 22 females) in the face mask plus hand hygiene group, 11 index cases (median age 7 years (IQR: 5-10); 5 males, 6 females) and 31 household contacts (median age 37 years (IQR: 12-43); 15 males, 16 females) in the face mask group and 13 index cases (median age 8 years (IQR: 7-10); 5 males, 8 females) and 36 household contacts (median age 35 years (IQR: 18-40); 18 males, 18 females) in the usual practices continuation group.  Index cases were defined as patients with a positive rapid antigen test for influenza (later to be confirmed by PCR), presenting to the study site within 2 days of symptom onset. These patients had to be at least 2 years old, had to be the only household member suffering from respiratory disease within 14 days prior to symptom onset. | Intervention:  Face mask plus hand hygiene program:  Households were provided with alcohol-based hand rub and surgical face masks and with information on the proper use of the interventions, through instructions by telephone, written information and demonstration by personnel.  Control:  Face mask program:  Households were provided with surgical face masks and information on their correct use, through instructions by telephone, written information and demonstration by personnel.  Extra control:  Usual practices continuation:  No masks or hand rub was provided.  [Data for the usual practices continuation group were not extracted.] | Secondary cases were defined as household members with influenza (laboratory-confirmed by PCR), a fever and a cough or sore throat.  (ILI (as defined by the WHO: fever and a cough or a sore throat) was the secondary outcome.)  Households received 4 or 5 visits during the observation period of 8 days. A nasal wash specimen (or nasal swab) was collected on each visit from all of the participating household members.  “Daily questionnaires to examine adherence and tolerability of the interventions” were used.  ICC: An intracluster correlation coefficient of 0.3 was assumed for sample size estimation in the context of secondary attack rates of laboratory confirmed influenza infection (Suess 2012).  ICC = 0.3 was used for the adjusted cluster-RCT analysis for both the influenza and ILI outcome (this review).  [Pooled data of the two face mask program groups and pooled data from the two influenza seasons were not extracted.] |
| **Interepidemic** | | | | | | |
| Aiello, 2010 | USA  (HIC) | Interepidemic | Experimental: Cluster-randomized controlled trial. | During the 2006–2007 influenza season (November 2006 to March 2007), university residence halls in Michigan, USA, with 1437 young adults were randomly assigned to either the face mask plus hand hygiene group (1 residence hall with 367 participants, mean age 18.6 (SD: 0.8), 188 males, 179 females, were analysed), face mask only group (4 residence halls with 378 participants, mean age 18.7 (SD: 0.8), 148 males, 230 females, were analysed), or usual practices continuation group (2 residence halls with 552 participants, mean age 18.4 (SD: 0.9), 100 males, 452 females, were analysed). The intervention lasted 6 weeks. | Intervention:  Face mask plus hand hygiene program:  Participants received packets of mask supplies in mailboxes, plus an educational video about standard medical procedure face masks and proper hand hygiene and additionally received bottles of alcohol-based hand sanitizer and written materials detailing appropriate hand sanitizer use.  Control:  Face mask program only:  Participants received packets of mask supplies in mailboxes, plus an educational video about standard medical procedure face masks.  Extra control:  No intervention. Usual practices continuation.  [Data for the usual practices continuation group were not extracted.] | ILI assessed via surveys and confirmed by influenza A/B testing (using PCR).  ILI case definition: presence of cough and at least 1 constitutional symptom (fever, chills, or body aches).  ICC: negative correlations were set to zero.  ICC = 0 was used for the adjusted cluster-RCT analysis (this review).  [Data on incidence of ILI were not extracted.]  [Data from self-reports of compliance (total mask hours per day, frequency of alcohol-based hand sanitizer use and health and hand hygiene practices) were not extracted.]  The 6 week-program excluded spring break. |
| Aiello, 2012 | USA  (HIC) | Interepidemic | Experimental: Cluster-randomized controlled trial. | During the 2007–2008 influenza season (November 2007 to March 2008), 37 residence houses in 5 university residence halls in Michigan, USA, with 1178 young adults were randomly assigned to either the face mask plus hand hygiene group (12 residence houses with 349 participants, mean age 19.01 (SD: 0.9), 168 males, 179 females, were analysed), face mask only group (13 residence houses with 392 participants, mean age 18.95 (SD: 1.0), 166 males, 225 females, were analysed), or usual practices continuation group (12 residence houses with 370 participants, mean age 18.90 (SD: 0.9), 162 males, 207 females, were analysed). The intervention lasted 6 weeks. | Idem ‘Aiello, 2010’. | Idem ‘Aiello, 2010’. |
| Biswas, 2019 | Bangladesh  (LMIC) | Interepidemic | Experimental: Cluster-randomized controlled trial. | 24 primary schools in Dhaka, Bangladesh (children aged 5–10 years) were randomized over the hand hygiene program group (12 schools, 5,077 students enrolled, on average 200 males per school and 223 females) and the control group (12 schools, 5,778 students enrolled, on average 223 males per school and 256 females) and the study was conducted in the influenza season, June–September of 2015, during a 10-week period. | Intervention:  Hand hygiene program:  Respiratory hygiene education, involving training teachers to convey intervention messages three times per week during hygiene education classes and in addition the placement of alcohol-based hand sanitizer on-site (each classroom and in toilets).  Control:  No intervention. Usual practices continuation.  However, at the end of the study period, educational materials (messages on handwashing with soap and respiratory hygiene etiquette) were provided. | Information on the number of ILI episodes was collected by phone if children were absent or by school visits by the study personnel, every other day. In case of ILI episodes, nasal swabs were collected from the child within 48 hours of symptom onset to detect influenza A or B (using standard PCR procedures).  ILI case definition: fever ≥38°C or subjective fever and cough.  [Data from structured observation of behavior related to handwashing and respiratory hygiene at school were not extracted.]  ICC: not reported, but sample size calculation in the context of ILI as an outcome was done accounting for a design effect (DE) of 1.5 (Biswas 2019).  DE = 1.5 was used for the adjusted cluster-RCT analysis (this review).  [Data on number of ILI episodes (and related absenteeism) were not extracted.]  [This study excluded the 6-week school vacation period from the 3^rd^ week of June to the 3^rd^ week of July from analysis.] |
| Cowling, 2008 | Hong Kong  (HIC) | Interepidemic | Experimental: Cluster-randomized controlled trial. | Hong Kong households with 198 index subjects, randomized from February to September 2007 to either the hand hygiene program group (32 households and 92 household contacts (37 males, 55 females) were followed up; 30 households and 84 household contacts were analysed), control group (74 households and 213 household contacts (83 males, 130 females) were followed up; 71 households and 205 household contacts were analysed) or face mask program group (22 households and 65 household contacts (26 males, 39 females) were followed up; 21 households and 61 household contacts were analysed), receiving home visits at 3, 6 and 9 days after the initial home visit.  [Mean ages not reported.]  Index case definition: index cases presented with ILI of <48 hours duration and were subsequently tested positive for influenza (using the QuickVue Influenza A+B rapid test). | Intervention:  Hand hygiene program:  Educational program about healthy diet and lifestyle and additionally a hand hygiene education program about the potential efficacy of proper hand hygiene in reducing transmission, including a demonstration of proper hand washing and hand antisepsis, plus the provision of automatic alcohol-based hand sanitizer, liquid hand soap to each household and individual small bottles of alcohol-based hand gel.  Control:  Educational program about healthy diet and lifestyle.  Extra intervention:  Face mask program:  Educational program about healthy diet and lifestyle and additionally a face mask education program, including a demonstration of proper face-mask wearing, plus the provision of a box with surgical face masks to each household member.  [Data for the face mask program (‘Extra intervention’) were not extracted.] | ILI definition: fever ≥37.8°C plus cough and/or sore throat (WHO definition).  The secondary attack rate (SAR) was measured as the proportion of household contacts of an index case who subsequently became ill with influenza (confirmed using standard laboratory PCR or viral culture).  ICC: the within-household correlation was 0.18 for the laboratory-confirmed SAR (Cowling 2008).  ICC = 0.18 was used for the adjusted cluster-RCT analysis (this review).  [Soap use (quantified by weighing) was not extracted, since this outcome was only measured in the intervention group and could therefore not be compared to the control group.]  [Data on self-reported outcomes (influenza, ILI) were not extracted.] |
| Cowling, 2009 | Hong Kong  (HIC) | Interepidemic | Experimental: Cluster-randomized controlled trial. | 259 Hong Kong households with 407 index subjects, randomized in a 1:1:1 ratio from January 2^nd^ through September 30^th^ 2008 to either the hand hygiene program group (85 households and 257 household contacts (median age 40 (IQR: 28-49), 103 males, 154 females) were analysed), control group (91 households, and 279 household contacts (median age 38 (IQR: 26-45), 105 males, 174 females) were analysed) or face mask plus hand hygiene program group (83 households and 258 household contacts (median age 38 (IQR: 27-48), 98 males, 160 females) were analysed), receiving home visits at 3 and 6 days after the initial home visit.  Index case definition: index cases presented with ILI of <48 hours duration and were subsequently tested positive for influenza (using the QuickVue Influenza A+B rapid test). | Intervention:  Hand hygiene program:  Educational program about healthy diet and lifestyle and additionally a hand hygiene education program about the potential efficacy of proper hand hygiene in reducing transmission, including a demonstration of proper hand washing and hand antisepsis, plus the provision of liquid hand soap for each kitchen and each bathroom to each household and individual small bottles of alcohol-based hand gel.  Control:  Educational program about healthy diet and lifestyle.  Extra intervention:  Face mask plus hand hygiene program:  Educational program about healthy diet and lifestyle and additionally a face mask education program, including a demonstration of proper face-mask wearing, plus the provision of a box with surgical face masks to each household member and a hand hygiene education program about the potential efficacy of proper hand hygiene in reducing transmission, including a demonstration of proper hand washing and hand antisepsis, plus the provision of liquid hand soap for each kitchen and each bathroom to each household and individual small bottles of alcohol-based hand gel.  [Data for the face mask plus hand hygiene program (‘Extra intervention’) were not extracted.] | ILI definition: fever ≥37.8°C plus cough and/or sore throat (WHO definition).  The secondary attack rate (SAR) was measured as the proportion of household contacts infected with influenza (confirmed with nasal or throat swab specimen positive for influenza by PCR).  ICC: within-household correlations of 0.12 for the RT-PCR–confirmed secondary attack ratios were used (Cowling 2009).  ICC = 0.12 was used for the adjusted cluster-RCT analysis (this review).  [Liquid hand soap use and alcohol-based hand rub (quantified by weighing) was not extracted, since this outcome was only measured in the intervention group and could therefore not be compared to the control group.]  [Data on self-reported outcomes (influenza, ILI, adherence) were not extracted.] |
| Stebbins, 2011 | USA  (HIC) | Interepidemic | Experimental: Cluster-randomized controlled trial. | 10 interested elementary schools were selected (3360 children) in Pittsburgh, USA and randomized to either the Pittsburgh Influenza Prevention Project (PIPP), a hand hygiene program (5 schools, 1695 people followed-up to determine cause of absence) or the control group (5 schools, 1665 people followed-up to determine cause of absence), from November 2007 through April 2008.  [Age and gender not reported.] | Intervention:  Hand hygiene program:  Schools received training in hand hygiene and respiratory hygiene (including a 45 min presentation regarding influenza) as part of the “WHACK the Flu” program and “refresher” trainings were implemented in January 2008. Students were encouraged to use alcohol-based hand sanitizer dispensers, placed on-site (in each classroom and all major common areas of intervention schools).  Control:  Usual hand hygiene practices continuation. No hand sanitizer dispensers were provided. | ILI case definition: fever ≥38°C with sore throat or cough  Primary outcome was an absence episode associated with ILI, that was subsequently laboratory confirmed as influenza A or B and influenza A H1 and H3 subtypes (using standard PCR procedures).  Influenza testing of absent students with ILI was performed only during the influenza season, of which the start was determined to be January 7^th^ 2008.  ICC: a coefficient of variation of the true rates between clusters within each group was assumed to be 0.1.  ICC = 0.1 was used for the adjusted cluster-RCT analysis (this review).  [Hand sanitizer use was not extracted, since this outcome was only measured in the intervention group and could therefore not be compared to the control group.]  [ILI cases, absenteeism due or not due to illness and adherence data were not extracted.] |
| Suess, 2012 | Germany  (HIC) | Epidemic and interepidemic | Experimental: Cluster-randomized controlled trial. | 84 households with an influenza positive index case (28 in the face mask plus hand hygiene group, 26 in the face mask group and 30 in the control group) and 218 household contacts (67 household contacts in the face mask plus hand hygiene group, 69 in the face mask group and 82 in the usual practices continuation group) were recruited for the study that was conducted during two consecutive influenza seasons (the pandemic season November 2009-January 2010 and January-April 2011) in Berlin, Germany.  In the 2010/2011 season there were 11 index cases (median age 7 years (IQR: 5-9); 7 males, 4 females) and 28 household contacts (median age 35 years (IQR: 15-43); 16 males, 12 females) in the face mask plus hand hygiene group, 15 index cases (median age 8 years (IQR: 4-9); 10 males, 5 females) and 38 household contacts (median age 35 years (IQR: 17-42); 19 males, 19 females) in the face mask group and 17 index cases (median age 8 years (IQR: 6-11); 13 males, 4 females) and 46 household contacts (median age 38 years (IQR: 12-43); 21 males, 25 females) in the usual practices continuation group.  Index cases were defined as patients with a positive rapid antigen test for influenza (later to be confirmed by PCR), presenting to the study site within 2 days of symptom onset. These patients had to be at least 2 years old, had to be the only household member suffering from respiratory disease within 14 days prior to symptom onset. | Intervention:  Face mask plus hand hygiene program:  Households were provided with alcohol-based hand rub and surgical face masks and with information on the proper use of the interventions, through instructions by telephone, written information and demonstration by personnel.  Control:  Face mask program:  Households were provided with surgical face masks and information on their correct use, through instructions by telephone, written information and demonstration by personnel.  Extra control:  Usual practices continuation:  No masks or hand rub was provided.  [Data for the usual practices continuation group were not extracted.] | Secondary cases were defined as household members with influenza (laboratory-confirmed by PCR), a fever and a cough or sore throat.  (ILI (as defined by the WHO: fever and a cough or a sore throat) was the secondary outcome.)  Households received 4 or 5 visits during the observation period of 8 days. A nasal wash specimen (or nasal swab) was collected on each visit from all of the participating household members.  “Daily questionnaires to examine adherence and tolerability of the interventions” were used.  ICC: An intracluster correlation coefficient of 0.3 was assumed for sample size estimation in the context of secondary attack rates of laboratory confirmed influenza infection (Suess 2012).  ICC = 0.3 was used for the adjusted cluster-RCT analysis for both the influenza and ILI outcome (this review).  [Pooled data of the two face mask program groups and pooled data from the two influenza seasons were not extracted.] |
| Talaat, 2011 | Egypt  (LMIC) | Interepidemic | Experimental: Cluster-randomized controlled trial. | 60 elementary government schools in Cairo were randomly selected. 30 schools (20,882 students enrolled (282,832 student-weeks of observation)) received the hand hygiene program over a 12-week period (February 16 to May 12, 2008) and 30 schools (23,569 students enrolled (250,584 student-weeks)) were in the control group.  [Age and gender info per group are not reported. However, “no significant differences were found for the 2 groups in median age (8 years) or sex distribution (51 % male)”.] | Intervention:  Hand hygiene program:  Campaign material for students, teachers and parents (including educational posters, booklets, informational flyers, …) and health messages through several hand hygiene activities for which a hand hygiene team, composed of 3 teachers, ensured that they were implemented at least once per week. Soap was either provided by parents, usually sending children to school a small bag containing bar soap and a clean towel or, if families could not afford soap and hand-drying material, the school administration provided them.  Control:  Usual hand hygiene practices continuation: handwashing, if done at all, was only performed by rinsing hands in water. Hands were typically dried on clothing or air-dried. | Before the campaign, neither soap nor hand-drying material was available in the schools. Handwashing, if done at all, was only performed by rinsing hands in water. Hands were typically dried on clothing or air-dried.  Hygiene teams interviewed parents of children, absent due to illness, by telephone to complete a survey on absenteeism and specific symptoms of illness.  Influenza cases: a nasal swab was taken from children (visiting the school clinic with ILI) and tested using QuickVue detection test for influenza A and B.  ICC: not reported. Design effect (DE) also not reported.  DE = 1.5 was used for the adjusted cluster-RCT analysis (this review), since the study characteristics from Biswas 2019 were most similar to Talaat 2011 (both clustering at the school level with school children in LMIC countries).  [Data on absenteeism due to diarrhea or conjunctivitis were not extracted.]  [Data for influenza cases in subgroup analyses per 4 weeks or per influenza subtype were not extracted.] |

LMIC: low- to middle-income country, HIC: high-income country, ICC: intra-cluster correlation coefficient, DE: design effect, ILI: influenza-like illness, PCR: polymerase chain reaction, RCT: randomized controlled trial, IQR: interquartile range, SD: standard deviation
