# Supplementary material for: The effect of hand hygiene promotion programs during epidemics and pandemics of respiratory droplet-transmissible infections on health outcomes: a rapid systematic review
Source: BMC Public Health. 2021 Sep 25;21:1745. doi: 10.1186/s12889-021-11815-4 (PMC8467175; doi:10.1186/s12889-021-11815-4)
Supplement: Supplementary file 4 — Additional file 4. Summary of findings. [file 12889_2021_11815_MOESM4_ESM.docx]

**Additional file 4: Summary of findings**

| **Epidemic** | | | | |
| --- | --- | --- | --- | --- |
| **Outcome** | **Comparison** | **Effect Size** | **#studies, total # participants per # clusters** | **Reference** |
| **Influenza positive cases (adjusted)** | | | | |
| Influenza positive cases | Hand hygiene program vs control | Meta-analysis  (see Fig. 2A)  Not statistically significant:  §  RR: 1.23, 95%CI [0.88;1.73] ¥  I² = 0%  (p=0.23)* | 2, 469/143 vs 552/155 | Ram, 2015 Simmerman, 2011 |
| Fig. 2A:  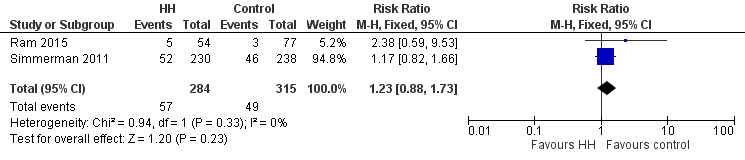 | | | | |
| Influenza positive cases | Facemask plus hand hygiene program vs control (facemask program) | Not statistically significant:  2/27 vs 2/21  RR: 0.78, 95%CI [0.12;5.07] ¥  (p=0.7928)* | 1, 39/17 vs 31/11 § | Suess, 2012 |
|  | | | | |
| **Other health-related outcomes (adjusted)** | | | | |
| Influenza-like-illness (ILI) cases | Hand hygiene program vs control | Meta-analysis  (see Fig. 2B)  Statistically significant:  RR: 1.56, 95%CI [1.14;2.14]  I² = 58%  (p=0.006)*  *In favour of control* | 2, 1953/312 vs 1800/303 | Ram, 2015  Simmerman, 2011 |
| Fig. 2B:  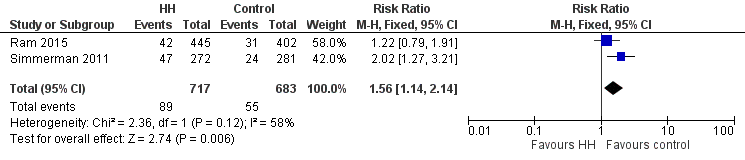 | | | | |
| Influenza-like-illness (ILI) cases | Facemask plus hand hygiene program vs control (facemask program) | Not statistically significant:  2/27 vs 3/21  RR: 0.52, 95%CI [0.10;2.83] ¥  (p=0.4478)* | 1, 39/17 vs 31/11 § | Suess, 2012 |
|  | | | | |
| **Outcome** | **Comparison** | **Effect Size** | **#studies, # participants** | **Reference** |
| **Other health-related outcomes (unadjusted)** | | | | |
| Number of absence episodes due to (physician-confirmed) ILI  (sick days/present days) | Alcohol-based hand gel every 60 min  vs control | Statistically significant:  0.017 vs 0.026  RD: 0.0096, 95%CI  [0.004;0.016]  (p=0.002)  *In favour of alcohol-based hand gel every 60 min* | 1, 452 vs 540 | Pandejpong, 2012 |
|  | Alcohol-based hand gel every 120 min  vs control | Not statistically significant:  0.025 vs 0.026  RD: 0.001, 95%CI  [-0.005;0.007]  (p=0.743) | 1, 447 vs 540 |  |
|  | Alcohol-based hand gel every 60 min  vs every 120 min | Statistically significant:  0.017 vs 0.025  RD: 0.009, 95%CI  [0.002;0.015]  (p=0.008)  *In favour of alcohol-based hand gel every 60 min* | 1, 452 vs 447 |  |
| Number of reported respiratory infection episodes (per total reported weeks) | Hand hygiene program (with soap and water) vs control | Not statistically significant:  507/6503 vs 474/6323  RaR: 1.04, 95%CI [0.92;1.17]  (p=0.5228)* | 1, 109 vs 119 § | Savolainen-Kopra, 2012 |
|  | Hand hygiene program (with alcohol-based hand rub) vs control | Statistically significant:  482/5672 vs 474/6323  RaR: 1.13, 95%CI [1.00;1.28]  (p=0.0433)*  *In favour of control* | 1, 97 vs 119 § |  |
|  | | | | |
| **Hand hygiene behavior (unadjusted)** | | | | |
| Number of hand washing episodes per day after 7 days | Hand hygiene program vs control | Statistically significant:  4.7 vs 3.9  MD: 0.8 £†  (p=0.002) | 1, [exact number of participants and clusters not reported for this analysis] | Simmerman, 2011 |
| Self-reported hand disinfection after coming home | Facemask plus hand hygiene program vs control (facemask program) | Statistically significant:  28/32 vs 11/23  RR: 1.83, 95%CI [1.17;2.86]  (p=0.0080)*  *In favour of facemask plus hand hygiene program* | 1, 32 vs 23 §  (household contacts)  [exact number of clusters not reported for this analysis] | Suess, 2012 |
| Self-reported hand disinfection after touching objects |  | Statistically significant:  28/32 vs 11/23  RR: 1.83, 95%CI [1.17;2.86]  (p=0.0080)*  *In favour of facemask plus hand hygiene program* |  |  |
| Self-reported hand disinfection before eating |  | Not statistically significant:  28/32 vs 23/23  RR: 0.88, 95%CI [0.76;1.02]  (p=0.0955)* |  |  |
| Self-reported hand disinfection after coughing/sneezing |  | Not statistically significant:  22/38 vs 17/30  RR: 1.02, 95%CI [0.68;1.55] ¥  (p=0.9192)* |  |  |

MD: mean difference, RR: risk ratio, RD: rate difference, RaR: rate ratio, SD: standard deviation, CI: confidence interval

* Calculations done by the reviewer(s) using Review Manager software

£ No raw data/SD’s available, CI cannot be calculated

¥ Imprecision (large variability of results)

† Imprecision (lack of data)

§ Imprecision (limited sample size or low number of events)

| **Interepidemic** | | | | |
| --- | --- | --- | --- | --- |
| **Outcome** | **Comparison** | **Effect Size** | **#studies, total # participants per # clusters** | **Reference** |
| **Influenza positive cases (adjusted)** | | | | |
| Influenza positive cases | Hand hygiene program vs control | Meta-analysis  (Fig. 3A)  Not statistically significant:  §  RR: 0.65, 95%CI [0.36;1.17] ¥  I² = 3%  (p=0.15)* | 2, 341/115 vs 484/162 | Cowling, 2008  Cowling, 2009 |
| Fig. 3A:  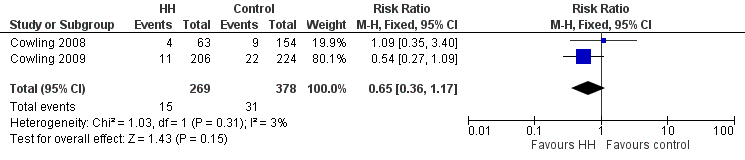 | | | | |
| Influenza positive cases | Hand hygiene program vs control | Meta-analysis  (Fig. 3B)  Statistically significant:  RR: 0.49, 95%CI [0.38;0.62]  I² = 0%  (p<0.00001)*  *In favour of hand hygiene program* | 3, 27654/47 vs 31012/47 | Biswas, 2019  Stebbins, 2011  Talaat, 2011 |
| Fig 3B:  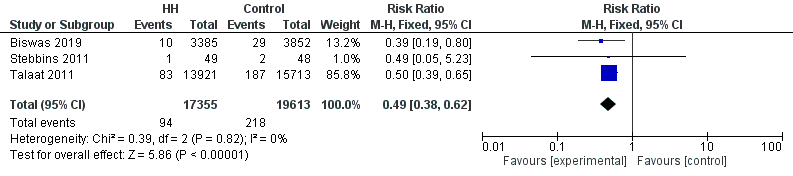 | | | | |
|  |  |  |  |  |
| Influenza positive cases | Facemask plus hand hygiene program vs control (facemask program) | Not statistically significant:  5/19 vs 2/26  RR: 3.42, 95%CI [0.74;15.79] ¥  (p=0.1150)* | 1, 28/11 vs 38/15 § | Suess, 2012 |
|  |  | Meta-analysis  (Fig. 3C)  Not statistically significant:  §  RR: 0.52, 95%CI [0.22;1.19] ¥  I² = 0%  (p=0.12)* | 2, 716/13 vs 770/17 | Aiello, 2010  Aiello, 2012 |
|  |  |  |  |  |
| Fig. 3C:  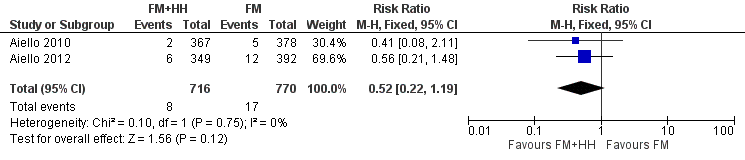 | | | | |
|  | | | | |
| **Hand hygiene behavior (unadjusted)** | | | | |
| Self-reported hand disinfection after coming home | Facemask plus hand hygiene program vs control (facemask program) | Not statistically significant:  20/22 vs 21/30  RR: 1.30, 95%CI [0.99;1.70] ¥  (p=0.0568)* | 1, 22 vs 30 §  (household contacts)  [exact number of clusters not reported for this analysis] | Suess, 2012 |
| Self-reported hand disinfection after touching objects |  | Not statistically significant:  16/22 vs 16/30  RR: 1.36, 95%CI [0.89;2.08] ¥  (p=0.1491)* |  |  |
| Self-reported hand disinfection before eating |  | Not statistically significant:  15/22 vs 27/30  RR: 0.76, 95%CI [0.56;1.03] ¥  (p=0.0786)* |  |  |
| Self-reported hand disinfection after coughing/sneezing |  | Not statistically significant:  8/28 vs 16/38  RR: 0.68, 95%CI [0.34;1.36] ¥  (p=0.2736)* | 1, 28 vs 38 §  (household contacts) |  |

RR: risk ratio, CI: confidence interval

* Calculations done by the reviewer(s) using Review Manager software

¥ Imprecision (large variability of results)

§ Imprecision (limited sample size or low number of events)
