# Supplementary material for: The effect of hand hygiene promotion programs during epidemics and pandemics of respiratory droplet-transmissible infections on health outcomes: a rapid systematic review
Source: BMC Public Health. 2021 Sep 25;21:1745. doi: 10.1186/s12889-021-11815-4 (PMC8467175; doi:10.1186/s12889-021-11815-4)
Supplement: Supplementary file 5 — Additional file 5. Cluster information. [file 12889_2021_11815_MOESM5_ESM.docx]

**Additional file 5: Cluster information**

| **Reference** | **Comparison** | **#events per #individuals (unadjusted)** | **Cluster setting** | **#clusters** | **ICC** | **DE** | **#events per #individuals (adjusted)** |
| --- | --- | --- | --- | --- | --- | --- | --- |
|  | | | | | | | |
| **Epidemic** | | | | | | | |
| **Influenza** | | | | | | | |
| Ram, 2015 | HH vs control | 17/177 vs 10/250 | Household contacts susceptible for influenza | 24 vs 36 | 0.37 | 3.26 | 5/54 vs 3/77 |
| Simmerman, 2011 |  | 66/292 vs 58/302 |  | 119 vs 119 | 0.18 | 1.27 | 52/230 vs 46/238 |
| Suess, 2012 | FM+HH vs FM | 3/39 vs 3/31 |  | 17 vs 11 | 0.30 | 1.45 | 2/27 vs 2/21 |
| **ILI** | | | | | | | |
| Ram, 2015 | HH vs control | 158/1661 vs 115/1498 | Household contacts susceptible for ILI | 193 vs 184 | 0.37 | 3.73 | 42/445 vs 31/402 |
| Simmerman, 2011 |  | 50/292 vs 26/302 | Household contacts susceptible for influenza | 119 vs 119 | 0.05 | 1.07 | 47/272 vs 24/281 |
| Suess, 2012 | FM+HH vs FM | 3/39 vs 4/31 |  | 17 vs 11 | 0.30 | 1.45 | 2/27 vs 3/21 |
| **Interepidemic** | | | | | | | |
| **Influenza** | | | | | | | |
| Cowling, 2008 | HH vs control | 5/84 vs 12/205 | Household contacts susceptible for influenza | 30 vs 71 | 0.18 | 1.34 | 4/63 vs 9/154 |
| Cowling, 2009 |  | 14/257 vs 28/279 |  | 85 vs 91 | 0.12 | 1.25 | 11/206 vs 22/224 |
| Biswas, 2019 |  | 15/5077 vs 43/5778 | Schoolchildren followed-up | 12 vs 12 | [NA]* | 1.50 | 10/3385 vs 29/3852 |
| Stebbins, 2011 |  | 51/1695 vs 53/1665 |  | 5 vs 5 | 0.10 | 34.50 | 1/49 vs 2/48 |
| Talaat, 2011 |  | 125/20882 vs 281/23569 |  | 30 vs 30 | [NA]** | 1.50 | 83/13921 vs 187/15713 |
| Suess, 2012 | FM+HH vs FM | 7/28 vs 3/38 | Household contacts susceptible for influenza | 11 vs 15 | 0.30 | 1.46 | 5/19 vs 2/26 |
| Aiello, 2010 |  | 2/367 vs 5/378 | University students analyzed | 1 vs 4 | 0.00 | 1.00 | 2/367 vs 5/378 |
| Aiello, 2012 |  | 6/349 vs 12/392 |  | 12 vs 13 | 0.00 | 1.00 | 6/349 vs 12/392 |

HH: hand hygiene, FM: facemask

* ICC not reported, but paper accounted for DE of 1.5.

** ICC not reported, but based on a similar paper by Biswas et al. (both performed in low-to-middle-income countries and clustering at the school level).
