# Supplementary material for: The effect of hand hygiene promotion programs during epidemics and pandemics of respiratory droplet-transmissible infections on health outcomes: a rapid systematic review
Source: BMC Public Health. 2021 Sep 25;21:1745. doi: 10.1186/s12889-021-11815-4 (PMC8467175; doi:10.1186/s12889-021-11815-4)
Supplement: Supplementary file 6 — Additional file 6. Risk of bias assessment and summary. [file 12889_2021_11815_MOESM6_ESM.docx]

**Additional file 6: Risk of bias assessment and summary**

| **Author, Year** | **Lack of allocation concealment** | **Lack of blinding** | **Incomplete accounting of outcome events** | **Selective outcome reporting** | **Other limitations** |
| --- | --- | --- | --- | --- | --- |
| **Epidemic** | | | | | |
| Pandejpong, 2012 | Lack of randomization: Unclear, randomization method not specified in the article.  Lack of allocation concealment: Unclear, not specified in the article. | Participants: Yes, blinding not possible.  Personnel: Yes, blinding not possible.  Outcome assessors: Unclear, not specified in the article. | No, no loss to follow-up. | No, all relevant outcomes were reported. |  |
| Ram, 2015 | Lack of randomization: No, “the list of random assignments was generated by an investigator with no contact with the human subjects”.  Lack of allocation concealment: No, “the field research officer consulted the block randomization list to make the assignment”. | Participants: Yes  Personnel: Yes  Outcome assessors: Yes  “Given the provision of a handwashing station as part of the intervention, it was not possible to ensure blinding of participants, intervention staff, or data collectors” | No, after randomization of households, only 2% withdrew or was excluded and less than 5% of household contacts did not have complete syndromic surveillance data. | No, all relevant outcomes were reported. | In 2009, index case-patients were recruited with symptom onset within 7 days preceding enrolment.  In 2010, index case-patients with symptom onset were recruited within 48 hours preceding enrolment, resulting in separate analyses for 2009 and 2010. |
| Savolainen-Kopra, 2012 | Lack of randomization: Unclear, randomization method not reported.  Lack of allocation concealment: Unclear, not reported. | Participants: Yes  Personnel: Yes  Outcome assessors: Yes  “The interventions were not blinded to any party involved (i.e. the study group, participants or the occupational health services).” | Yes, only 76% of volunteers who started reporting continued to do so until the end of the study. | No, all relevant outcomes were reported. | The national hand hygiene campaign (in the context of the 2009 influenza emergence) could have influenced the control arm.  Therefore subanalyses were performed; for the period before the end of July, 2009 (25 weeks; “before the pandemic”), and thereafter until the end of May, 2010 (43 weeks; “during and after the pandemic”). |
| Simmerman, 2011 | Lack of randomization: Unclear. Randomization method is not reported.  Lack of allocation concealment: Unclear, a study coordinator assigned each household to one study arm after consent was obtained, but not clear whether concealed. | Participants: Yes, blinding not possible.  Personnel: Yes, blinding not possible.  Outcome assessors: No, recruiting clinicians were blinded to the allocation of the specific intervention. | No, the percentage of households that did not complete 7 days of follow-up was limited to <10% per group. | No, all relevant outcomes were reported. |  |
| Suess, 2012 | Lack of randomization: No, random numbers lists (MS Excel 2003) were used to randomly assign index cases to one of the three study groups.  Lack of allocation concealment: No, recruiting physicians were not aware of the allocation of the numbers to the interventions. | Participants:  Yes, blinding not possible.  Personnel: No.  Outcome assessors: No.  Physicians and laboratory personnel were blinded: intervention material was provided in closed boxes. | No, no loss to follow-up. | No, all relevant outcomes were reported. | There are differences between the two study seasons:  In 2009/2010 all viruses belonged to A (H1N1) pdm09, while in 2010/2011 both A (H1N1) pdm09 as well as B viruses circulated.  In 2010/2011 a significantly larger number of both index patients and household contacts was vaccinated compared to 2009/2010.  In 2010/2011 a higher proportion of households was visited by study personnel within 36 hours, compared to 2009/2010.  In the season 2009/2010, one of the control households reported wearing masks, another of the control households reported wearing masks and using alcohol based hand sanitizer. |
| **Interepidemic** | | | | | |
| Aiello, 2010 | Lack of randomization: No, “The residence hall units were randomized by blindly selecting a uniform ticket with the name of each hall out of a container.”  Lack of allocation concealment: Unclear, not specified in the article. | Participants: Yes, blinding not possible.  Personnel: Yes, blinding not possible.  Outcome assessors: Unclear, not specified in the article. | No, less than 10% of participants were lost to follow-up (99/1396). | No, all relevant outcomes were reported. | Influenza incidence was very low, only 94 samples (all three groups together) were obtained for PCR testing and only 10 of those were positive. |
| Aiello, 2012 | Lack of randomization: No, randomization was done by study staff using SAS software (Proc Plan) to each time select a random allocation sequence until all residences were allocated.  Lack of allocation concealment: Unclear, not specified in the article. | Participants: Yes, blinding not possible.  Personnel: Yes, blinding not possible.  Outcome assessors: No, “PI’s and statisticians were blinded to intervention status during analyses”. | No, less than 10% of participants were lost to follow-up (73/1184). | No, all relevant outcomes were reported. |  |
| Biswas, 2019 | Lack of randomization: No, a computer-based randomization method was used.  Lack of allocation concealment: Unclear, not specified in the article. | Participants: Yes  Personnel: Yes  Outcome assessors: Yes  “The study team, schoolchildren, their parents, and teachers were not blinded to the intervention.” | No, “as the intervention was only for 10 weeks and in the middle of the academic year, there was no dropout.” | No, all relevant outcomes were reported. | ILI (and not laboratory-confirmed influenza) was the outcome used for power analyses.  Due to a limited budget, samples were not collected for all students. Only 10 to 12% of the enrolled students were tested for influenza and the samples were only collected within 72 hours of symptom onset for the control group, whereas this was done within 48 hours for the intervention group.  Observed handwashing may have increased more in intervention schools than in control schools, as the children in the intervention schools knew they were supposed to be washing their hands.  We do not know how much of the benefit may have been due to the altered coughing or sneezing behavior and how much was due to improved hand hygiene. |
| Cowling, 2008 | Lack of randomization:  No, randomization lists were prepared by a biostatistician using a random number generator (R software).  Lack of allocation concealment:  No, “The allocation to specific intervention arms was concealed to recruiting doctors/clinics throughout.” | Participants: Yes  Personnel: Yes  Outcome assessors: Unclear, not specified in the article.  “Participants and those administering interventions were not blinded to the interventions.” | Yes, dropout was higher than anticipated; despite being advised of the study requirements and although they gave informed consent before being recruited into the study (and tested by rapid influenza test without charge), 35% of randomized subjects/households refused to allow any home visits. | No, all relevant outcomes were reported. | “This pilot study was not powered to detect small or moderate efficacies of the interventions with statistical significance … Lessons learnt from this pilot have informed changes for the main study in 2008.” |
| Cowling, 2009 | Lack of randomization:  No, randomization lists were prepared by a biostatistician using a random number generator (R software).  Lack of allocation concealment:  No, “The  allocation to specific intervention groups was concealed to  recruiting physicians and clinics throughout the study.” | Participants: Yes  Personnel: Yes  Outcome assessors: Unclear, not specified in the article.  “Participants and people who administered the interventions were not blinded to the interventions.” | Yes, “After random assignment, 76 (19%) of the households declined home visits or could not be contacted after numerous repeated attempts.” | No, all relevant outcomes were reported. | “The delay from index patient symptom onset to intervention and variable adherence may have mitigated intervention effectiveness.” |
| Stebbins, 2011 | Lack of randomization: No, interested schools were selected and randomized using a SAS macro for constrained randomization.  Lack of allocation concealment: No, “The random allocation to two arms was created by Dr. Cummings and concealed until intervention assignment.” | Participants: Yes  Personnel: Yes  Outcome assessors: Yes  “Blinding of assignment to the intervention or control group was not possible because of the nature of the intervention.” | Yes, follow-up of absence episodes was limited. The cause of absence was only determined in approx. one third of the episodes (1087/3179 for intervention group and 1158/3390 for control).  Of the ILI episodes, only between one fourth and one fifth was tested for influenza (41/171 for intervention group and 41/190 for control). | No, all relevant outcomes were reported. |  |
| Suess, 2012 | Lack of randomization: No, random numbers lists (MS Excel 2003) were used to randomly assign index cases to one of the three study groups.  Lack of allocation concealment: No, recruiting physicians were not aware of the allocation of the numbers to the interventions. | Participants:  Yes, blinding not possible.  Personnel: No.  Outcome assessors: No.  Physicians and laboratory personnel were blinded: intervention material was provided in closed boxes. | No, no loss to follow-up. | No, all relevant outcomes were reported. | There are differences between the two study seasons:  In 2009/2010 all viruses belonged to A (H1N1) pdm09, while in 2010/2011 both A (H1N1) pdm09 as well as B viruses circulated.  In 2010/2011 a significantly larger number of both index patients and household contacts was vaccinated compared to 2009/2010.  In 2010/2011 a higher proportion of households was visited by study personnel within 36 hours, compared to 2009/2010.  In the season 2009/2010, one of the control households reported wearing masks, another of the control households reported wearing masks and using alcohol based hand sanitizer. |
| Talaat, 2011 | Lack of randomization: Unclear, school selection is at random, but whether the allocation to intervention vs control groups was at random is not stated.  “60 elementary schools (30 intervention and 30 control schools) were randomly selected from a numbered list of all 725 government elementary schools in Cairo by using a computer-generated random number table.”  Lack of allocation concealment: Unclear, not specified in the article. | Participants:  Yes  Personnel:  Yes  Outcome assessors:  Yes  “Study teams and schoolchildren and their parents were not blinded to the intervention.” | No, no loss to follow-up. | No, all relevant outcomes were reported. | “Low rate of testing in students who were absent because of ILI in the control schools compared to the intervention schools (12% vs 22%).”  “The use of rapid tests for diagnosis of laboratory-confirmed influenza with known low sensitivity (60% in some studies) likely resulted in an underestimation of illness in each group.” |

For the **epidemic** studies, 2 studies^19,23^ used appropriate random sequence generation and allocation concealment, whereas this was unclear for 3 other studies^18,20,21^. 4 out of 5 studies performed during an epidemic period^18-21^ did not have proper blinding of participants and personnel, whereas 1 study did have proper blinding^23^. On the other hand, 2 out of 5 studies did have proper blinding of the outcome assessors^21,23^, whereas this was unclear for 1 study^18^ and not properly done for 2 studies^19,20^. For none of the epidemic studies^18-21,23^ there was incomplete reporting of outcome data or selective outcome reporting, except for one study^20^ in which we found that there was a risk of attrition bias, due to incomplete outcome data. There was risk of other bias for one study^20^, and no risk of other bias for 2 other studies^18,21^, whereas this was unclear for another 2 studies^19,23^.

For the **interepidemic** studies, 7 studies^13-17,22,23^ used appropriate random sequence generation, whereas this was unclear for one other study^24^. 4 out of 8 of these studies used appropriate allocation concealment^16,17,22,23^ and this was not clear for the rest of the studies^13-15,24^. Only one study performed proper blinding of participants and personnel^23^, whereas this was not clear for one other study^16^ and not performed for the 6 other studies^13-15,17,22,24^. Blinding of outcome assessors was properly done in 2 studies^14,23^, unclear for 3 studies^13,16,17^ and not performed for the 3 other studies^15,22,24^. There was incomplete reporting of outcome data in 3 studies^16,17,22^, whereas there was no risk of attrition bias for the 5 other studies^13-15,23,24^. Lastly, none of the interepidemic studies^13-17,22-24^ selectively reported their data, resulting in no risk of reporting bias. There was risk of other bias for 4 studies^13,15,17,24^, and no risk of other bias for 2 other studies^14,22^, whereas this was unclear for another 2 studies^16,23^.
